# Urinary and Serum Metabolomics Analyses Uncover That Total Glucosides of Paeony Protect Liver against Acute Injury Potentially via Reprogramming of Multiple Metabolic Pathways

**DOI:** 10.1155/2017/9038260

**Published:** 2017-12-11

**Authors:** Haojie Li, Wenli Cao, Mengxi Lu, Chunxiao Wu, Xinguo Wang, Liying Niu

**Affiliations:** ^1^Shijiazhuang Hospital of Traditional Chinese Medicine, Shijiazhuang, Hebei 050051, China; ^2^Hebei TCM Formula Granule Engineering & Technology Research Center, Hebei University of Chinese Medicine, Shijiazhuang, Hebei 050200, China; ^3^North China University of Science and Technology, Tangshan, Hebei 063210, China; ^4^Hebei Hospital of Traditional Chinese Medicine, Shijiazhuang, Hebei 050011, China

## Abstract

Total glucosides of paeony (TGP) have been confirmed to be hepatoprotective. However, the underlying mechanism is largely unclear. In this study, we investigated the metabolic profiles of urine and serum in rats with carbon tetrachloride- (CCl_4_-) induced experimental liver injury and TGP administration by using ultra-performance liquid chromatography-mass spectrometry (UPLC-MS). The vehicle or a single dose of TGP was intragastrically administered to Wistar rats once a day for 14 consecutive days. To induce ALI, 50% CCl_4_ was injected intraperitoneally into these rats 2 hours after the last time administration of saline of TGP at the 14th day. The results indicated that TGP administration could protect rats from CCl_4_-induced ALI and alanine aminotransferase (ALT) and aspartate aminotransferase (AST) elevation, as well as hepatocyte apoptosis and inflammation. Furthermore, metabolomics analysis showed that TGP treatment significantly attenuated CCl_4_-triggered deregulation of multiple metabolites in both urine and serum, including glycine, alanine, proline, and glutamine. Metabolite set enrichment and pathway analyses demonstrated that amino acid cycling and glutathione metabolism were two main pathways involved in CCl_4_-induced experimental liver injury and TGP administration. Taken together, these findings revealed that regulation of metabolites potentially plays a pivotal role in the protective effect of TGP on ALI.

## 1. Introduction

Sudden hepatocyte damage induced by drugs, hepatitis virus infections, hepatic ischemia reperfusion, and toxins often causes ALI, which represents a common pathological basis of various liver diseases [1, 2]. Long-term liver injury often leads to liver fibrosis and hepatocellular carcinoma, which are life-threatening conditions associated with high morbidity and mortality [3]. Therefore, the prevention of ALI occurrence and progression is essential for clinical treatment of liver diseases. Appropriate balance between cell death and compensatory cell proliferation is critical for normal physiological functions of liver. Imbalance of hepatocyte death and compensatory proliferation plays a pivotal role in the outcome of ALI. However, the underlying mechanisms are still only poorly understood.

Carbon tetrachloride (CCl_4_) is a manufactured chemical mainly used in the production of chlorofluorocarbons. Previous studies have shown that CCl_4_ is a classical reagent for induction of ALI, which has been used for many years to investigate the mechanisms involved in acute and chronic liver injury and to screen hepatoprotective drugs [4–6]. A short-term treatment of CCl_4_ may lead to hepatocyte necrosis and steatosis, while prolonged administration may induce liver fibrosis, and even hepatocellular carcinoma (HCC). CCl_4_ can enhance cellular oxidative stress and recruit inflammatory cells and therefore leads to hepatic architectural damage and functional failure. The CCl_3_ and CCl_3_O_2_ radicals generated by CCl_4_ impair hepatocytes directly by activation of microsomal cytochrome P450 and covalently binding to macromolecules in liver cells and attack unsaturated lipids under the cytoplasmic membrane to induce lipid peroxidation (LPO) and alter the permeability of the plasma, lysosomal, and mitochondrial membranes. This process is followed by the release of inflammatory cytokines and activation of resident hepatic macrophages (Kupffer cells), which are thought to potentiate ALI [5, 7, 8].

As a biologically active compound extracted from* Paeonia lactiflora* pall root, the total glucosides of paeony contain paeoniflorin, hydroxyl-paeoniflorin, paeonin, albiflorin, and benzoylpaeoniflorin and are widely used in China for pain relief and treating rheumatoid arthritis (RA), systemic lupus erythematosus (SLE), and liver diseases [9–11]. The currently accepted mechanism of TGP treatment of ALI is that TGP presents anti-inflammatory, antioxidative, and immunoregulatory activities, with few side effects. Gonzalez et al. showed that TGP attenuated inflammation and ROS. Meanwhile, TGP inhibited hydrogen peroxide (H_2_O_2_) released from peritoneal macrophages in adjuvant arthritis (AA) rats and had beneficial effects on hepatic fibrosis in rats by inhibition of collagen synthesis and decreasing oxidative stress [12].

As an emerging “-omics” science in systems biology and a powerful approach to investigate cellular metabolic reprogramming associated with the disease state, metabolomics, particularly mass spectrometry (MS) based metabolomics, aims to identify low-molecular-weight metabolites in tissue cells and body fluids in response to disease progression. It can help us to identify the potential biomarkers of disease and discover the mechanism involved in disease formation and progression. Clinically, metabolomics offers a potential approach to clinical diagnosis and predictive value for the therapeutic intervention of disease. Some metabolomics studies have been applied to ALI. For instance, according to UPLC-MS/MS based serum metabolomics analysis in experimental animal models, Gonzalez et al. found that several metabolites including glucose, amino acids, and membrane lipids were significantly modified with a high correlation with the degree of liver damage [12]. In conclusion, this study supports that UPLC-MS/MS based serum metabolomics in experimental animal models could be a powerful approach to search for biomarkers in liver injury. Wang et al. applied metabolomics approach to study the influence of Radix Paeoniae Alba and Radix Paeoniae Rubra on the metabolic changes in rats with acute liver injury and identified several potential biomarkers including creatine, deoxycholic acid, choline, 5-methylenetetrahydrofolate, folic acid, and glycocholic acid [13].

In this study, metabolomics profiling was performed by using UPLC-MS to compare the difference of serum metabolic profiles in CCl_4_-induced ALI model rats after administration of vehicle or TGP. Our results indicated the protective effect of TGP against liver injury. In addition, several potential biomarkers including glycine, alanine, and proline and glutamine were detected and identified. Metabolite set enrichment and pathway analyses demonstrated that amino acid cycling and glutathione metabolism were two main pathways involved in CCl_4_-induced experimental liver injury and TGP administration. These metabolic changes suggest that metabolic changes may play an important role in the protective effect of TGP against ALI.

## 2. Materials and Methods

### 2.1. Chemical Reagents and Medicines

Methanol, acetonitrile, and methanoic acid were purchased from Fisher-Scientific (Fair Lawn, NJ, USA). CCl_4_ and corn oil (analytical grade) were purchased from the Nanjing Chemical Reagent Co., Ltd. (Nanjing, China). UltraPure DNase/RNase-free distilled water from Invitrogen (Carlsbad, CA, USA) was used throughout the study. Total glucosides of paeony (TGP) were prepared by our lab as described in our previous paper [14] and the extraction yields were 42.64% and 15.20% for paeoniflorin and albiflorin, respectively.

### 2.2. Animal Maintenance and Experiments

Thirty male Wistar rats (6 weeks, male, 260 g ± 20 g) were obtained from the Laboratory Animal Center of the Hebei University of Chinese Medicine. All animal experiments were performed with the approval of local ethical committee of Hebei University of Chinese Medicine. The rats were maintained in a specific pathogen free laboratory with a temperature of 24 ± 2°C under a 12 h light/dark cycle and allowed food and water ad libitum. The animals were acclimatized in our laboratory for one week before the experiments were performed. Rats were randomly divided into 3 groups, 10 animals for each group. Group 1, the control group, was given intragastrical administration of saline once a day for 14 consecutive days and intraperitoneal injections of corn oil (10 ml/kg body weight) 2 hours after the last time administration of saline at the 14th day. Group 2, the ALI group, was given intragastrical administration of saline once a day for 14 consecutive days and intraperitoneal injections of 50% CCl_4_ (CCl_4_ : corn oil = 1 : 1) (10 ml/kg body weight) 2 hours after the last time administration of saline at the 14th day. Group 3, the ALI plus TGP group, was given intragastrical administration of TGP (1.41 g/kg) once a day for 14 consecutive days and intraperitoneal injections of 50% CCl_4_ (CCl_4_ : corn oil = 1 : 1) (10 ml/kg body weight) 2 hours after the last time administration of TGP at the 14th day.

### 2.3. H&E Staining

The left lobes of liver tissues for histopathological analysis were fixed in 10% paraformaldehyde. Fixed tissues were then embedded in paraffin, sectioned at 5 *μ*m thickness, and stained with hematoxylin and eosin (H&E). The protocol for H&E staining was as follows: the wax block was placed in a −20°C freezer 30 min before sectioning. Then the tissues were sliced up and the sections were baked at 70°C overnight, dewaxed, hydrated in distilled water, stained with hematoxylin for 5 min, differentiated in hydrochloric acid alcohol, blued in ammonia water, counterstained with eosin for 10 s, dehydrated with ethanol at different concentrations (75%, 90%, and 100% ethanol), transparentized with xylene twice, and finally mounted in neutral gum. The sections were observed under a microscope.

### 2.4. Determination of ALT and AST Levels

Serum alanine aminotransferase (ALT) and aspartate transaminase (AST) levels were measured with an automated chemistry analyzer (ThermoFisher Scientific).

### 2.5. Urine and Serum Sample Preparation

Urine samples were collected from the metabolic cages after fasting for 24 h and centrifuged (10 min, 4000 rpm, 4°C). Then the supernatant was filtrated using 0.45 *μ*m membrane (Millipore, German). The filtrate was dissolved in acetonitrile and precipitated overnight, and solution was centrifuged (10 min, 12000 rpm, 4°C). The supernatant was collected and stored at −80°C for further analysis. Blood samples were collected from the retro-orbital sinus from anesthetized rats with sterile tubes. Then the fresh blood samples were stayed at 4°C for 30 min and centrifuged at 3000 rpm for 15 min. Proteins were precipitated from the serum samples by adding three volumes of acetonitrile in 1.5 ml microtubes at room temperature. After brief vortex mixing the samples were kept overnight at −20°C. Supernatants were collected after centrifugation at 12000 rpm, 4°C for 10 min, transferred to sterile tube, and stored at −80°C for UPLC®-MS analysis.

### 2.6. UPLC-MS/MS Analysis

Chromatography was performed on a 2.1 × 100 mm Hypersil Gold aQ C18 (ThermoFisher Scientific, Waltham, MA, USA) using an LC-100 system (Wufeng, Shanghai, China). The mobile phase consisted of 0.1% methanoic acid in methanol (mobile phase A) and acetonitrile containing 0.1% formic acid (mobile phase B). The volume of sample injected onto the column was 5 *μ*l. A gradient elution at a flow of 0.30 ml/min was performed with an initial composition of 15% A, which was held for 3.5 min, followed by an increase in 0.01 min to 100% A (for 1.5 min) and finally a reequilibration (5 min). The total run time was 10 min. The eluent was introduced into the mass spectrometer (AB SCIEX Q-Triple 4500 System, AB SCIEX, Redwood City, California, USA) by electrospray ionization, with capillary and cone voltages set in the positive and negative ion modes to 3,200 and 30 V, and 2,800 and 50 V, respectively. The nebuliser gas was set to 600 l/h at a temperature of 350°C. The cone gas was set to 50 l/h and the source temperature was set to 150°C. Centroid data were acquired from* m/z* 50–850 using an accumulation time of 0.2 s per spectrum. Data were acquired and processed using Markerview 1.2.1 software (AB SCIEX).

### 2.7. Multivariate Data Analysis

The partial least-squares discrimination analysis (PLS-DA) was further performed with the unit-variance scaled UPLC-MS/MS data as *X* matrix and class information as *Y* matrix to identify the metabolites that significantly contribute to intergroup differentiation. The PLS-DA models were validated using a sevenfold cross validation method and the quality of the model was described by the parameters of *R*2*X* and *Q*2 values. The Variable Importance in the Projection (VIP) value (VIP  > 1) was used to evaluate the variable contribution and identify the potential biomarkers. Metabolite set enrichment analysis was performed by using online software MetaboAnalyst (http://www.metaboanalyst.ca/).

### 2.8. Statistical Analysis

The univariate statistical analysis was performed by SPSS Statistics 20.0 (Armonk, New York, United States) and *p* value was set as 0.05 for statistical significance.

## 3. Results

### 3.1. TGP Protects Hepatocyte from CCl_4_-Induced ALI

**Figure 1:**
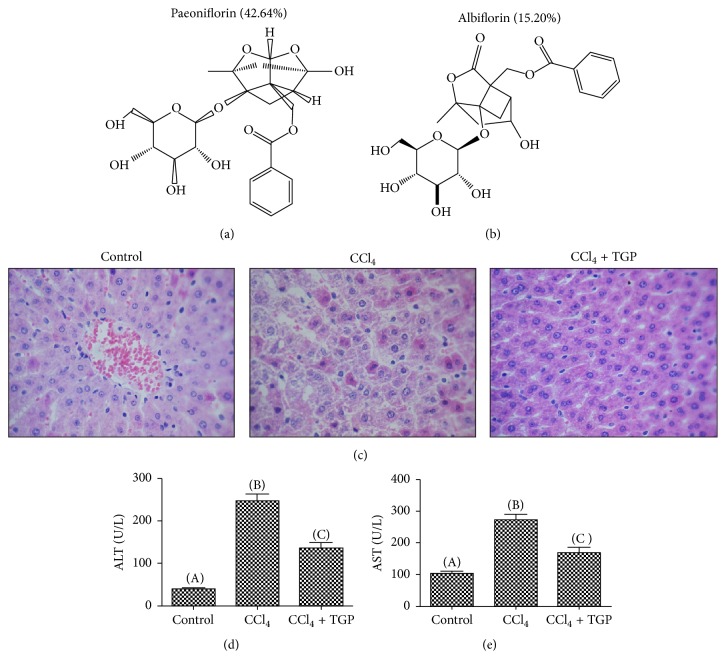
TGP protects hepatocytes from CCl_4_-induced ALI. ((a) and (b)) Chemical structures of paeoniflorin and albiflorin, which are the two main compounds in the TGP extracted by our laboratory. (c) H&E staining of liver tissues in the control, CCl_4_, and CCl_4_ plus TGP groups. ((d) and (e)) Evaluation of ALT (d) and AST (e) levels in serum samples obtained from the indicated groups. All the data were statistically analyzed by Student's *t*-test. Different letters above the bars indicate statistically significant difference with *p* < 0.05.

Firstly, to determine the efficacy of TGP extract (Paeoniflorin: 42.64%, Albiflorin: 15.20%, as shown in Figures 1(a) and 1(b)) on CCl_4_-induced ALI, the left lobes of liver tissue were collected from groups 1–3 and subjected to H&E staining. As shown in Figure 1(c), in the ALI model group, significant anomalies of liver cells and degeneration of structure were observed in CCl_4_ treated rats, including vacuolization of cytoplasm and infiltration of inflammatory cell in portal area. These phenomena were alleviated in the TGP-administrated group. In this group, the animal showed a relatively normal liver structure with minor cytoplasmic vacuolization and inflammatory cell infiltration. Meanwhile, compared to the control group, both the serum levels of ALT and AST significantly increased in the ALI group after CCl_4_ injection (Figures 1(d) and 1(e)). In addition, these serum makers from the TGP administration group significantly decreased compared with those in the ALI group (Figures 1(d) and 1(e)). Overall, these results suggested that TGP administration could effectively protect the liver from CCl_4_-induced acute liver damage.

### 3.2. Multivariate Analysis of UPLC-MS/MS Results of Urine and Serum: Discrimination between Control, CCl_4_, and CCl_4_ + TGP Groups

**Figure 2:**
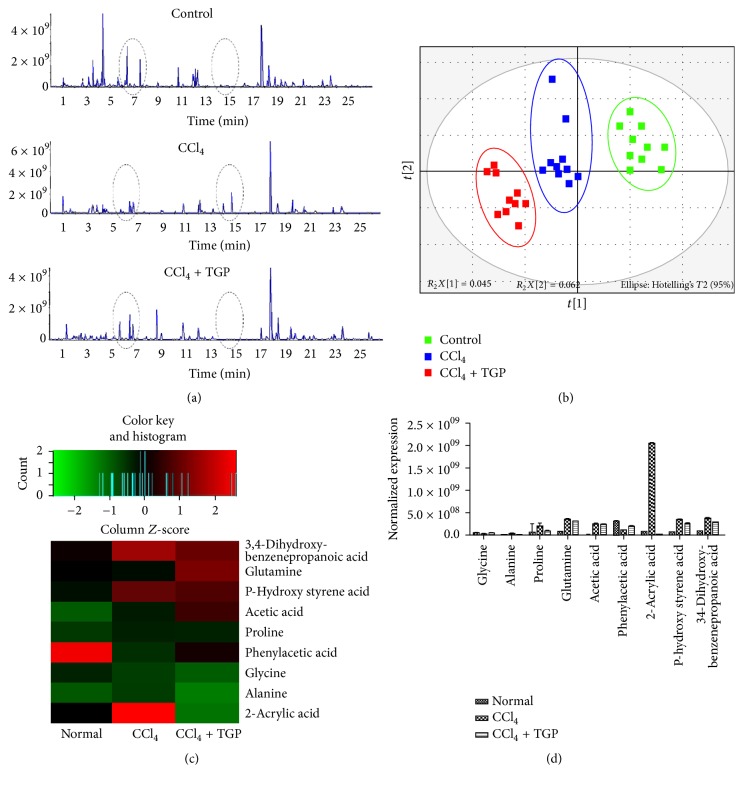
Metabolomics profiles and multivariate data analyses in urine. (a) The representative urine UPLC-MS TIC chromatograms in the indicated groups. (b) PLS-DA score plot for the control, CCl_4_, and CCl_4_ plus TGP groups. (c) Heatmap is shown from hierarchical clustering analyses of metabolomics changes in urine samples of the control, CCl_4_, and CCl_4_ plus TGP groups. (d) Normalized expression of discriminating metabolites in the indicated groups.

**Figure 3:**
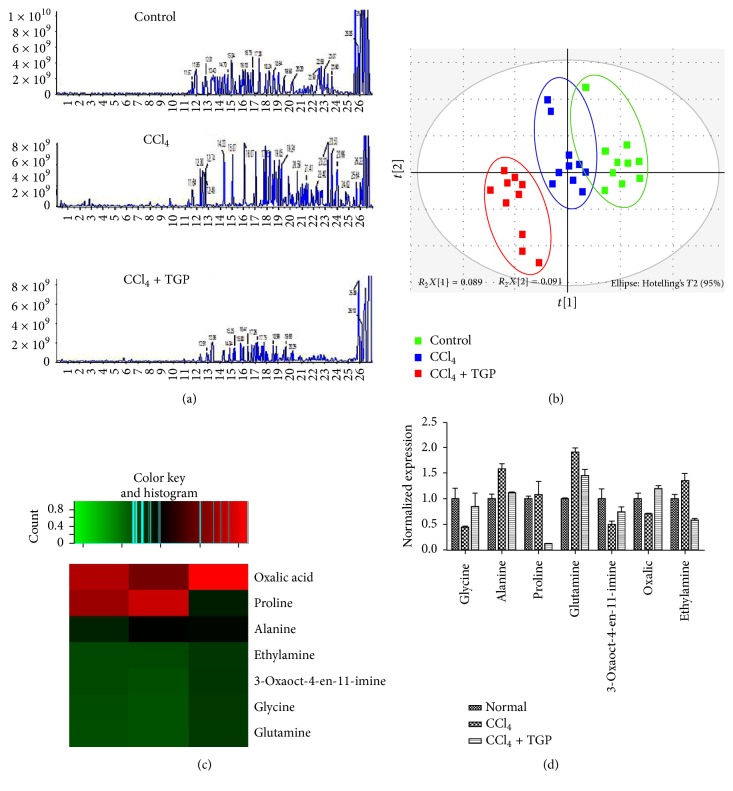
Metabolomics profiles and multivariate data analyses in serum. (a) The representative serum UPLC-MS TIC chromatograms in the indicated groups. (b) PLS-DA score plot for the control, CCl_4_, and CCl_4_ plus TGP groups. (c) Heatmap is shown from hierarchical clustering analyses of metabolomics changes in serum samples of the control, CCl_4_, and CCl_4_ plus TGP groups. (d) Normalized expression of discriminating metabolites in the indicated groups.

To further explore the metabolic events associated with protective effects of TGP on CCl_4_-induced ALI, serum samples were examined through UPLC-MS/MS based metabolomics analysis. The representative TIC chromatograms of urine and serum samples derived from the control, CCl_4_, and CCl_4_ + TGP groups were shown in Figures 2(a) and 3(a), respectively. The UPLC-MS/MS results for serum samples were then subjected to multivariate data analysis to unravel changes of the serum metabolic profiles in these samples. The scores of partial least-squares discriminant analysis (PLS-DA) for all groups were presented in Figures 2(b) and 3(b), respectively. The distinctive separation of serum samples collected at these samples indicated that CCl_4_ dramatically altered the chemical composition of serum and TGP administration showed a reversed effect (Figures 2(b) and 3(b)).

### 3.3. Differentially Expressed Metabolite Identification among the Control, CCl_4_, and CCl_4_ + TGP Groups

According to the UPLC-MS/MS results, a total of 9 discriminating metabolites (VIP > 1.0, *p* < 0.05) and 7 discriminating metabolites were identified in the urine and serum, respectively (Figures 2(c), 2(d), 3(c), and 3(d); Tables 1 and 2). These results showed that most metabolites increased in the CCl_4_-induced ALI samples, suggesting accelerated metabolism processes in this group. Correlation analysis between each discriminating metabolite level and the observed ALT or AST activity was performed both in urine and in serum (Tables 3 and 4). In urine, significant positive correlations were found in alanine, proline, and 2-acrylic acid compared with ALT and AST (Table 3). Meanwhile, obvious negative correlations were found in glycine and phenylacetic acid compared with ALT and AST (Table 3). In addition, in the serum samples, alanine and glutamine were positively correlated with ALT and AST, but glycine, 3-oxaoct-4-en-11-imine, oxalic acid, and ethylamine were negatively correlated with ALT, AST (Table 4). These findings indicate that alanine, downregulation of proline and 2-acrylic acid alanine and glutamine, and upregulation of glycine, phenylacetic acid, 3-oxaoct-4-en-11-imine, oxalic acid, and ethylamine may be potential biomarkers in the evaluation of the efficacy of TGP treatment on ALI.

### 3.4. The Metabolic Pathways Related to TGP Treatment in CCl_4_-Induced ALI

**Figure 4:**
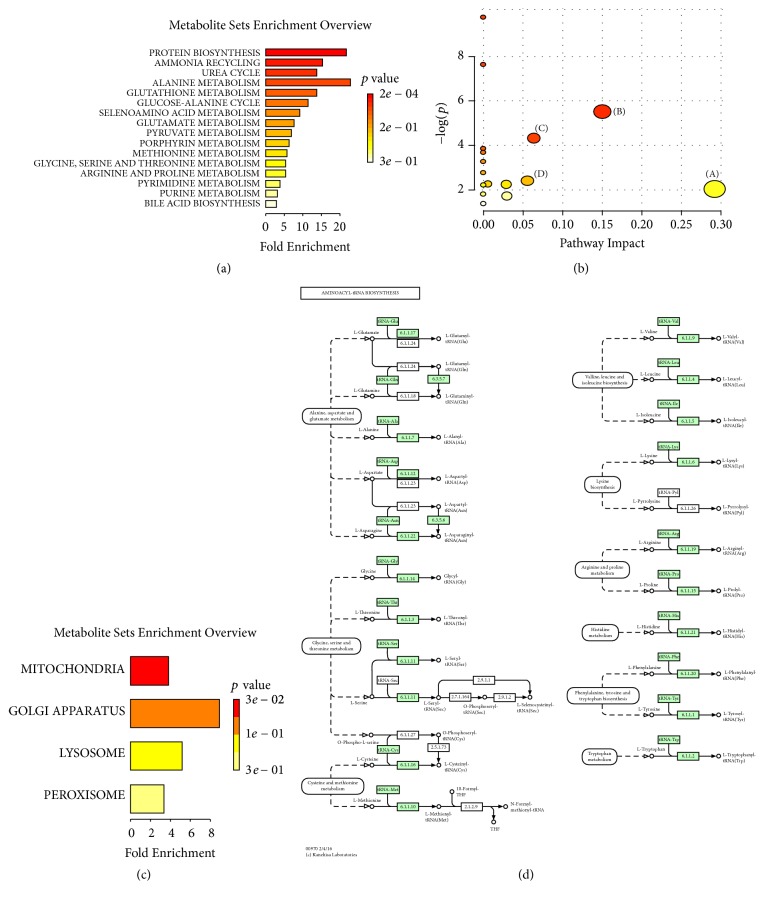
Metabolic pathways involved in TGP treatment of CCl_4_-induced ALI. (a) Metabolite set enrichment analysis (MSEA). (b) Pathway analysis. (c) Location-based metabolite sets. (d) Schematic representation of protein biosynthesis.

More detailed analysis of metabolite set enrichment and pathways influenced by TGP was performed by the online software MetaboAnalyst (http://www.metaboanalyst.ca/). The metabolite set enrichment was shown in Figure 4(a) and the pathways were shown in Figure 4(b). The most relevant pathways, such as protein biosynthesis and ammonia recycling urea cycle, were identified. Location-based metabolite sets analysis demonstrated that these metabolic pathways were mainly related to the organelle mitochondria (Figure 4(c)), suggesting that energy metabolism plays a critical role in these processes. Generally, we can conclude that the amino acid cycling (Figure 4(d)) and glutathione metabolism were the two most important pathways influenced by TGP in CCl_4_-induced ALI.

## 4. Discussion

Traditional Chinese medicine formulas are an important source in the search for new drug candidates. Natural extracts from these formulas have contributed to the therapeutics to various diseases including ALI. However, the targets and underlying mechanisms of these compounds were not well elucidated. In this study, we have performed an UPLC-MS/MS analysis to detect candidate endogenous metabolites suitable for the evaluation of efficacy of TGP on ALI. Our study has demonstrated that TGP not only suppresses ALT, AST, inflammation, and cellular apoptosis but also reverses a series of significant metabolite changes such as glycine, alanine, proline, and glutamine in the CCl_4_-induced ALI model. The advantages of this study include the developed metabolomic strategy and advanced multivariate statistical analysis such as PLS-DA. Using a combination of these techniques, not only was the whole plasma metabolic profiling characterized, but also the urine metabolic profiling was investigated.

Liver tissue is the main site of amino acid metabolism. Hepatic damage will inevitably result in deregulation of amino acid cycling [15, 16]. Previous studies demonstrated that oxidation and overproduction of reactive oxygen species play a pivotal role in CCl_4_-induced ALI. Under the oxidation condition, tricarboxylic acid cycle was attenuated. Meanwhile, the glycine levels are related to glutathione production. In our study, the alanine and proline levels were increased in CCl_4_-induced ALI and the glycine level was decreased. As two important intermediates in energy metabolism, alanine and proline were highly associated with tricarboxylic acid cycle [15–17]. These results suggested that the two metabolic pathways, tricarboxylic acid cycle and glutathione production, may play important role in CCl_4_-induced ALI and TGP treatment. In previous study, metabolomics analyses have been applied in several ALI models. For instance, Beger et al. demonstrated that acylcarnitines, bile acids, and pyroglutamic acid could be potential markers related to APAP-induced liver injury [18]. Gonzalez et al. showed that the serum levels of sphingomyelins, amino acids (methionine, threonine, or tyrosine), and free fatty linoleic (18 : 2) acid were significantly altered in galN-induced ALI [12]. These results suggest that the metabolic changes in ALI may depend on different models. In our model, we found that the amino acid cycling and glutamine metabolism might play a critical role in CCl_4_-induced ALI and the efficacy of TGP. The function and underlying mechanism of TGP in other ALI models are necessary to be elucidated.

CCl_4_ is frequently accepted as a typical liver toxicant and the CCl_4_-induced hepatic injury model has been widely used to investigate the factors involved in regulation of hepatotoxicity. In CCl_4_-induced ALI model, increased levels of ROS induced by damage cells destroy the permeability of endoplasmic reticulum, mitochondria, and plasma membranes, which finally lead to leakage of various enzymes in liver tissues into the circulating system. Therefore, evaluation of liver-associated enzyme levels in the serum is a useful tool to assess the hepatocellular damage. In the present study, we found that serum ALT and AST levels were increased in CCl_4_-induced ALI model, and TGP significantly suppressed ALT and AST levels, indicating TGP can effectively eliminate the liver-associated enzymes in the circulating system. Obviously, the mechanisms of metabolites changes in these groups may include the deregulation of these enzymes in blood and urine. In the future, further studies are still needed to investigate the proteomics profiles of serum and urine samples in these groups.

One of the major challenges in investigating the underlying mechanisms of the hepatoprotective effects of TGP is how to evaluate the contributions of different active components in these processes. In future study, we plan to determine the protective roles of different glucosides in ALI and their underlying mechanisms.

## Figures and Tables

**Table 1:** Relative values of differentially expressed metabolites in urine derived from the control, CCl_4_, and CCl_4_ + TGP groups.

Metabolites	Control		CCl_4_		CCl_4_ + TGP	
	Mean	SD	Mean	SD	Mean	SD
Glycine	53046982.6	7600000	25885583.6	9144515.66	46879562.1	8246525.17
Alanine	4044630.7	550671.17	31061116.1	5506716.23	6167215.3	416823.13
Proline	55723727.6	202145548.4	202145548.4	74120374.1	92835647.2	4221364.19
Glutamine	85802389.2	2688625.98	344623827.2	24990531.2	315612382.2	2789521.16
Acetic acid	1212640.1	5898250.99	248909373.9	29612215.7	239913356.4	2154783.18
Phenylacetic acid	309182480.9	9778348.39	119431883.1	1059814.42	189372561.3	8658458.45
2-Acrylic acid	85802389.2	2688625.98	2036423827	24990531.2	17541387.8	2587785.57
P-Hydroxy styrene acid	71449906.4	5428615.98	344623299.2	14910521.2	261579865.2	4368714.48
3,4-Dihydroxy-benzenepropanoic acid	95583449.6	900792.62	376501012.1	2776860.74	294573579.6	806754.12

**Table 2:** Relative values of differentially expressed metabolites in serum derived from the control, CCl_4_, and CCl_4_ + TGP groups.

Metabolites	Control		CCl_4_		CCl_4_ + TGP	
	Mean	SD	Mean	SD	Mean	SD
Glycine	11802069.5	2453210.23	5145640.4	414633.93	9867552.2	3256721.14
Alanine	147284114.6	13379477.27	232970617.5	16104373.26	162651294.9	2324764.83
Proline	754538905.6	22346595.32	812346375.8	4184375.75	92835647.2	4221364.19
Glutamine	4289012.3	337658.85	8192843.8	1230976.4	6199283	1176549.4
3-Oxaoct-4-en-11-imine	26918264.2	1327698.88	13289102.8	29612215.7	20182732	3287699.58
Oxalic acid	826663219.5	87597632.77	571293743	29835445.7	991736434	54287345.3
Ethylamine	28374333.6	3429876.11	38297431.5	4758221.3	16394854.7	2350965.22

**Table 3:** Correlation analysis between the urine levels of differentially expressed metabolites and the activity of the aminotransferases, alanine aminotransferase (ALT), and aspartate aminotransferase (AST).

Metabolites	ALT		AST	
	*r*	*p*	*r*	*p*
Glycine	−0.425	0.026^*∗*^	−0.392	0.033^*∗*^
Alanine	0.661	0.007^*∗∗*^	0.548	0.009^*∗∗*^
Proline	0.440	0.011^*∗*^	0.414	0.014^*∗*^
Glutamine	0.084	0.433	0.066	0.496
Acetic acid	0.095	0.386	0.083	0.440
Phenylacetic acid	−0.481	0.018^*∗*^	−0.533	0.022^*∗*^
2-Acrylic acid	0.793	0.002^*∗∗*^	0.658	0.004^*∗∗*^
P-Hydroxy styrene acid	0.113	0.330	0.104	0.382
3,4-Dihydroxy-benzenepropanoic acid	0.148	0.249	0.122	0.288

^*∗*^ *p* < 0.05; ^*∗∗*^*p* < 0.01.

**Table 4:** Correlation analysis between the serum levels of differentially expressed metabolites and the activity of the aminotransferases, alanine aminotransferase (ALT), and aspartate aminotransferase (AST).

Metabolites	ALT		AST	
	*r*	*p*	*r*	*p*
Glycine	−0.483	0.021^*∗*^	−0.422	0.025^*∗*^
Alanine	0.336	0.038^*∗*^	0.305	0.044^*∗*^
Proline	0.140	0.301	0.114	0.312
Glutamine	0.459	0.029^*∗*^	0.463	0.025^*∗*^
3-Oxaoct-4-en-11-imine	−0.496	0.019^*∗*^	−0.467	0.026^*∗*^
Oxalic acid	−0.282	0.043^*∗*^	−0.339	0.053
Ethylamine	−0.387	0.036^*∗*^	0.349	0.039^*∗*^

^*∗*^ *p* < 0.05.

## Abbreviations

TCM: Traditional Chinese medicine
TGP: Total glucosides of paeony
ALI: Acute liver injury
CCL_4_: Carbon tetrachloride
ALT: Alanine aminotransferase
AST: Aspartate aminotransferase
PCA: Principal component analysis
PLS-DA: Partial least-squares discrimination analysis
VIP: Variable Importance in the Projection
HCC: Hepatocellular carcinoma
LPO: Lipid peroxidation
H_2_O_2_: Hydrogen peroxide
AA: Adjuvant arthritis
UPLC-MS: Ultra-performance liquid chromatography-mass spectrometry
RA: Rheumatoid arthritis
SLE: Systemic lupus erythematosus
H&E: Hematoxylin and eosin staining.
